# Stable *DNMT3L* overexpression in SH-SY5Y neurons recreates a facet of the genome-wide Down syndrome DNA methylation signature

**DOI:** 10.1186/s13072-021-00387-7

**Published:** 2021-03-09

**Authors:** Benjamin I. Laufer, J. Antonio Gomez, Julia M. Jianu, Janine M. LaSalle

**Affiliations:** 1 Department of Medical Microbiology and Immunology, School of Medicine, University of California, Davis, CA 95616 USA; 2 Genome Center, University of California, Davis, CA 95616 USA; 3 MIND Institute, University of California, Davis, CA 95616 USA

**Keywords:** DNMT3L, Down syndrome, Whole genome bisulfite sequencing, Bivalent chromatin, Epigenomics, PiggyBac transgenesis

## Abstract

**Background:**

Down syndrome (DS) is characterized by a genome-wide profile of differential DNA methylation that is skewed towards hypermethylation in most tissues, including brain, and includes pan-tissue differential methylation. The molecular mechanisms involve the overexpression of genes related to DNA methylation on chromosome 21. Here, we stably overexpressed the chromosome 21 gene *DNA methyltransferase 3L* (*DNMT3L*) in the human SH-SY5Y neuroblastoma cell line and assayed DNA methylation at over 26 million CpGs by whole genome bisulfite sequencing (WGBS) at three different developmental phases (undifferentiated, differentiating, and differentiated).

**Results:**

*DNMT3L* overexpression resulted in global CpG and CpG island hypermethylation as well as thousands of differentially methylated regions (DMRs). The *DNMT3L* DMRs were skewed towards hypermethylation and mapped to genes involved in neurodevelopment, cellular signaling, and gene regulation. Consensus *DNMT3L* DMRs showed that cell lines clustered by genotype and then differentiation phase, demonstrating sets of common genes affected across neuronal differentiation. The hypermethylated *DNMT3L* DMRs from all pairwise comparisons were enriched for regions of bivalent chromatin marked by H3K4me3 as well as differentially methylated sites from previous DS studies of diverse tissues. In contrast, the hypomethylated *DNMT3L* DMRs from all pairwise comparisons displayed a tissue-specific profile enriched for regions of heterochromatin marked by H3K9me3 during embryonic development.

**Conclusions:**

Taken together, these results support a mechanism whereby regions of bivalent chromatin that lose H3K4me3 during neuronal differentiation are targeted by excess DNMT3L and become hypermethylated. Overall, these findings demonstrate that *DNMT3L* overexpression during neurodevelopment recreates a facet of the genome-wide DS DNA methylation signature by targeting known genes and gene clusters that display pan-tissue differential methylation in DS.

**Supplementary Information:**

The online version contains supplementary material available at 10.1186/s13072-021-00387-7.

## Background

DS is the leading genetic cause of intellectual disability and results from trisomy 21 [1]. However, genes outside of chromosome 21 are also altered in DS and differences in gene expression and DNA methylation are observed across the entire genome. Most DS tissues exhibit differentially methylated sites that tend to be hypermethylated when compared to typically developing controls [2]. Furthermore, some genes show pan-tissue differential methylation, which is also skewed heavily towards hypermethylation. Mechanistically, there are a number of genes located on chromosome 21 that belong to pathways related to DNA methylation and have the potential to result in the hypermethylation pattern observed in DS [2]. Functional experimentation into the cause of hypermethylation has demonstrated a key role of the chromosome 21 encoded DNA methyltransferase *DNMT3L* at select genes [3]. While DNMT3L is catalytically inactive, it is a regulatory factor that binds to and stimulates the de novo methyltransferases DNMT3A and DNMT3B [4–6].

Structurally, DNMT3L and DNMT3A form elongated heterotetramers through their C-terminal domains (DNMT3L–DNMT3A–DNMT3A–DNMT3L), and this complex multimerizes on DNA to form nucleoprotein filaments that spread DNA methylation over larger regions, such as CpG islands [7–9]. Members of the de novo methyltransferase family (DNMT3A,B,L) contain an ATRX–DNMT3–DNMT3L (ADD) domain that binds to the unmodified histone H3 tail (H3K4me0), to localize DNA methylation to previously unmethylated regions, and this binding is inhibited by methylation at lysine 4 of H3 (H3K4me3) [10–12].

In mouse embryonic stem cells (mESCs), DNMT3L has been reported to be either a positive or negative regulator of DNA methylation depending on genomic context [13]; however, another report found it to only function as a positive regulator [14]. This difference appears to be due to the mESCs cell lines used as well as the methodologies used to assay DNA methylation, since the studies examined different regions of the genome at different levels of resolution. Experiments using transient *DNMT3L* overexpression in mESCs and somatic cells have demonstrated a role for DNMT3L in assembling a repressive chromatin modifying complex that silences retroviral sequences through de novo DNA methylation and methylation-independent mechanisms [15]. Finally, DNMT3L is also required for the establishment of parental imprinting patterns in developing gametes [16, 17].

While *DNMT3L* is a prime candidate for the genome-wide DS DNA hypermethylation profiles, the genome-wide effect of *DNMT3L* overexpression has not yet been characterized. Previously, we profiled post-mortem DS brain and compared to matched controls using WGBS. We observed a genome-wide wide impact on DNA methylation profiles, where ~ 75% of the differentially methylated regions (DMRs) were hypermethylated and mapped to genes involved in one-carbon metabolism, membrane transport, and neurotransmission. Given these observations, we sought to characterize the effect of *DNMT3L* overexpression on different phases of neuronal development by assaying differentiating SH-SY5Y cells.

SH-SY5Y cells are a human neuronal cell line that have been subcloned three times from the SK-N-SH cell line, which was derived from metastatic cells of a 4-year-old female with neuroblastoma. Unlike many other cancer lines, the SH-SY5Y cell line has a stable karyotype with a dominant ploidy of 2, which includes trisomy 1q and 7, gains on 2p and 17q, and losses on 14q and 22q [18–20]. Similar to other cancer cell lines, SH-SY5Y cells have a relatively hypomethylated genome when compared to neurons from human post-mortem brain samples [21, 22]. SH-SY5Y cells display a mixed morphology that is primarily neuroblast-like with short processes; however, there is also a smaller proportion of epithelial-like cells, which likely reflects the cell lines multipotency and an origin from the neural crest. SH-SY5Y cells can be consistently differentiated from a neuroblast-like state into a homogenous population of mature dopaminergic neurons in 18 days [23]. The differentiation protocol is a three-phased reprogramming approach, which involves gradual serum deprivation and the addition of retinoic acid during the first two phases and then a transition to an extracellular matrix where the cells are given a neuronal media that lacks serum. The neuron-like cells become dependent on neurotrophic factors, such as BDNF, for their survival and the epithelial-like cells, which require serum, do not survive. Notably, during this differentiation process, serial splitting via a brief incubation with low strength trypsin allows for the selection of the less adherent neuron-like cells (for the next phase or harvest) and ultimately results in a morphologically pure culture of mature dopaminergic neurons in the final phase. Thus, SH-SY5Y cells serve as an ideal model system to investigate the methylome-wide effects of *DNMT3L* overexpression on undifferentiated, differentiating, and differentiated human neurons.

Here, we interrogated the contribution of *DNMT3L*, a chromosome 21 encoded gene, in DS-associated genome-wide changes in DNA methylation. Stable overexpression of *DNMT3L* in human SH-SY5Y cells provided a model system to test the effects of this known methylation regulator on genome-wide DNA methylation across neuronal differentiation. We found that *DNMT3L* overexpression recreated a facet of the genome-wide DNA profile observed in DS tissues, including brain. Interestingly, chromatin state analyses revealed that the *DNMT3L*-induced hypermethylated DMRs were enriched within bivalent domains, whereas the hypomethylated DMRs were enriched within heterochromatin. These results indicate that an increase in *DNMT3L* copy number during neuronal differentiation leads to chromatin-specific effects on DNA methylation.

## Results

### Stable *DNMT3L* Overexpression in SH-SY5Y Cells

**Fig. 1:**
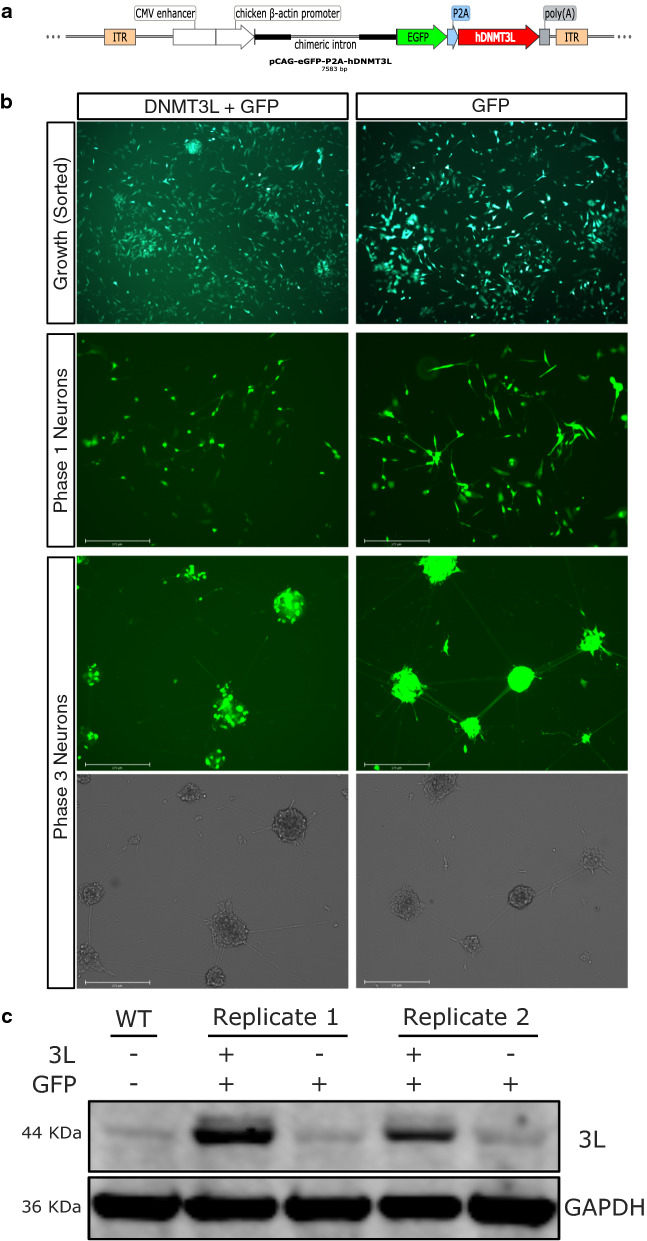
Stable overexpression of human *DNMT3L* in SH-SY5Y cells at different phases of neural differentiation. **a** PiggyBac vector for stable integration of human *DNMT3L* (*hDNMT3L*), which was co-expressed with *eGFP*. Upon translation the self-cleaving peptide (P2A) sequence between the polypeptide resulted in separate proteins. The vector was flanked by inverted terminal repeats (ITRs) that were recognized by the PiggyBac transposon. **b** Representative live cell images the *DNMT3L* + *eGFP* and *eGFP* cell lines during the different phases assayed in either the GFP or phase contrast channels. **c** Western blot of DNMT3L and GAPDH in wild-type SH-SY5Y cells (WT) and the transgenic cell lines

SH-SY5Y cells were transfected with either myc-tagged human *DNMT3L* [4] and *enhanced GFP* (*eGFP*) via a piggyBac transposase [24] construct or a matched control construct without *DNMT3L* (Fig. 1a). eGFP-positive cells were selected by flow cytometry and then cultured again to enrich for a cell line with stable *DNMT3L* overexpression. For the experimental investigations, three developmental phases were assayed: 1) undifferentiated cells (growth), 2) the first phase of differentiation (phase 1), and 3) the final phase of differentiation (phase 3) that represents the pure and mature neuronal culture (Fig. 1b). When compared to the growth phase, phase 1 neurons displayed a relatively elongated cell body with an increased number of longer neurites with neuronal growth cones that were starting to form synapses with their neighboring cells. Notably, phase 1 neurons are the most commonly assayed form of differentiated SH-SY5Y cells in the literature. Phase 2 is a relatively brief intermediate phase for the final selection that occurs in phase 3 and, thus, was not examined. After being plated as a monolayer, phase 3 neurons migrated to form large spheres of highly interconnected neurons, which were similar to neurospheres; however, they are were attached to an extracellular matrix and produced a three-dimensional network with a larger-scale organization that consists of long tracts of bundled processes. Notably, the eGFP-only control cells displayed visibly higher fluorescence levels than those with DNMT3L + eGFP, a result that was observed in all cell lines. Endogenous DNMT3L expression was confirmed by Western blot in all transfected cell lines and compared to a non-transfected cell line (WT). As expected, DNMT3L expression was much higher in the transgenic DNMT3L cell lines and only low basal levels were detected in the WT and eGFP-only control cell lines (Fig. 1c).

### Stable *DNMT3L* overexpression results in global and CpG island hypermethylation

**Fig. 2:**
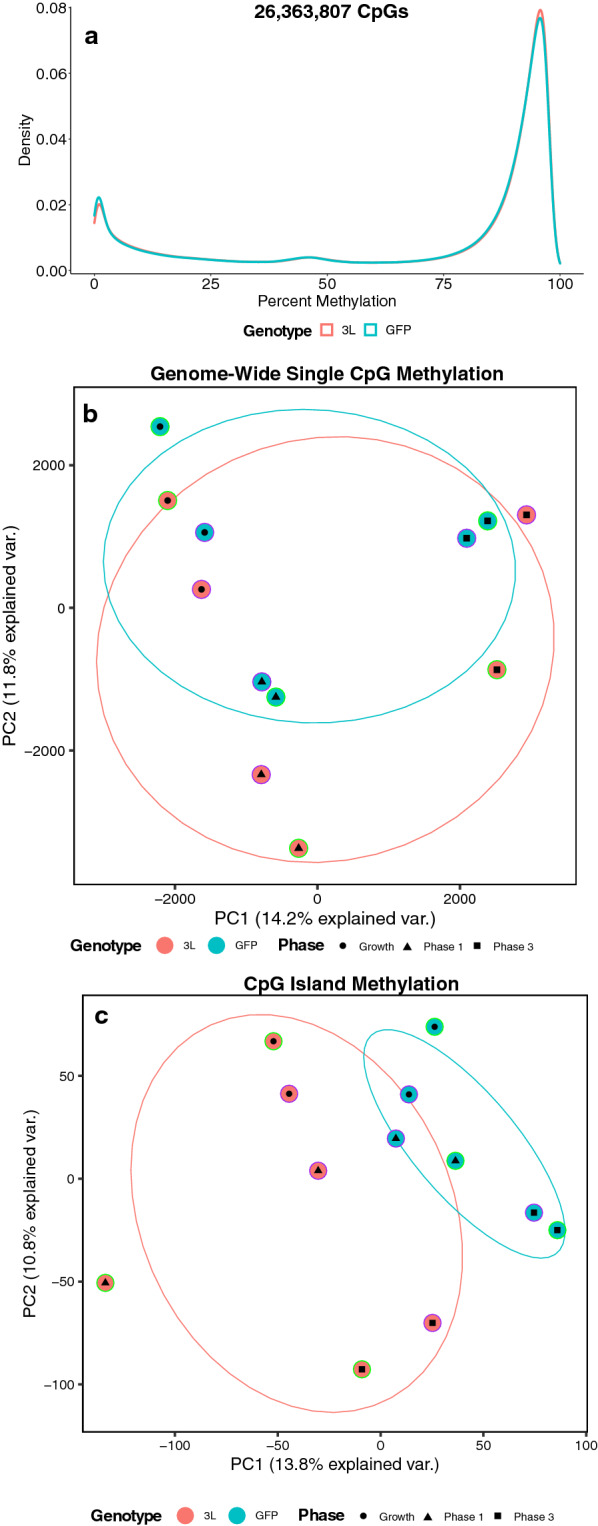
Global methylation profiles of *DNMT3L* overexpression. **a** Density plots of the mean of smoothed individual CpG methylation values for each cell line at the different phases. **b** Principal component analysis (PCA) of smoothed individual global CpG methylation values. **c** PCA of smoothed individual CpG island methylation values. For each PCA, the color of the outermost shape represents the cell line, where green represents the first batch of cell lines and purple represents the second. The ellipses represent the 68% confidence interval, which represents 1 standard deviation from the mean for data with a normal distribution

WGBS libraries were constructed using DNA isolated from all cell lines and sequenced to > 10 × coverage, resulting in > 26 million assayed CpGs. There was a significant (*p* = 0.016) increase in global CpG methylation (Fig. 2a) and a significant (*p* = 0.004) increase in global CpG island methylation in cells with *DNMT3L* overexpression (3L) when compared to vector-only (*eGFP*) controls. Cell developmental phase, herein referred to as “phase”, also had a significant (*p* = 0.004) effect on global CpG methylation and a significant (*p* = 0.030) effect on global CpG island methylation. In the growth phase, *DNMT3L* cells showed 69.70% global methylation and 34.11% CpG island methylation, while the *eGFP* cells showed 69.43 and 33.82%, respectively. In phase 1 of differentiation, *DNMT3L* cells showed 70.38% global methylation and 34.39% CpG island methylation, while *eGFP* cells showed 69.93 and 33.89%, respectively. In phase 3 of differentiation, the *DNMT3L* cells showed 69.68% global methylation and 33.95% CpG island methylation, while *eGFP* cells showed 69.33 and 33.52%, respectively. Notably, both global CpG methylation and global CpG island methylation levels were the highest in phase 1 and the lowest in phase 3. Principal component analysis (PCA) revealed that the cells clustered together by phase for both the 20 Kb window and single CpG approaches (Fig. 2b), but clustered by genotype for the CpG island approach (Fig. 2c).

### *DNMT3L* DMRs map to genes related to neurodevelopment, cellular signaling, and gene regulation

**Fig. 3:**
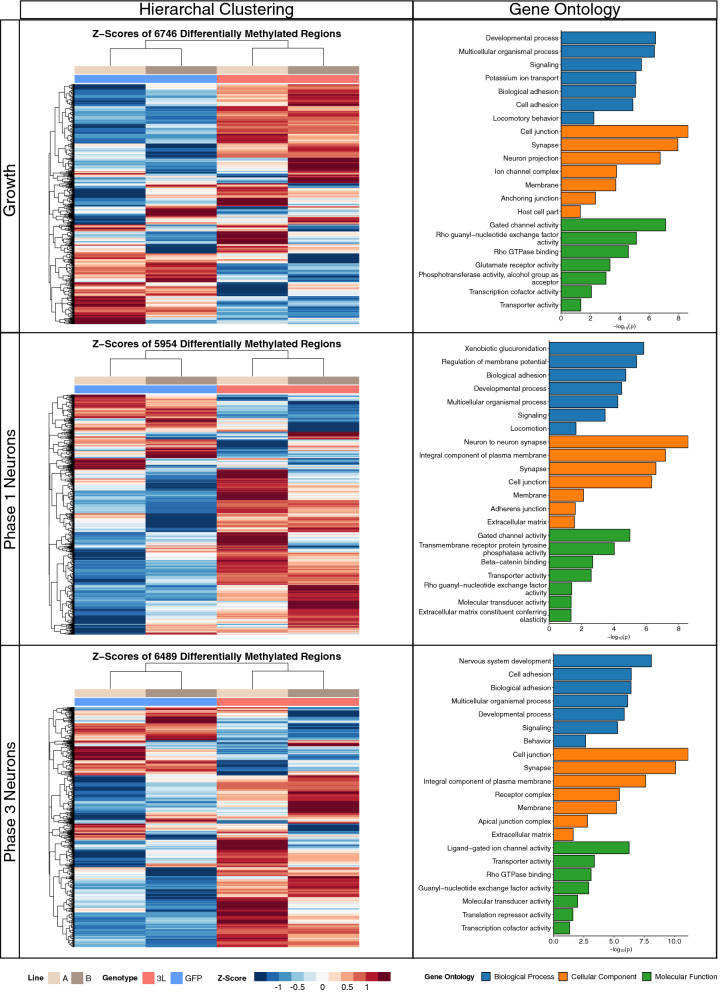
Differentially methylated region (DMR) profiles and slimmed gene ontology (GO) enrichments for *DNMT3L* overexpression at the different phases of neural differentiation. The heatmaps represent hierarchal clustering of Z-scores, which represent the number of standard deviations from the mean of the non-adjusted individual smoothed methylation value for each DMR. The bar plots represent the least dispensable significant enrichments (*p* < 0.05)

To examine the specific genetic loci altered by *DNMT3L* overexpression during neuronal differentiation, differentially methylated regions (DMRs) were identified using pairwise genotype comparisons of all three phases. The replicate cell lines were utilized as biological replicates and batch was directly adjusted for in the analyses. The significant (permutation *p* < 0.05) DMRs from each pairwise phase comparison were skewed towards hypermethylation (Additional File 1: Supplementary Table 1). In the growth phase, 62% of the 6746 DMRs were hypermethylated, as well as 71% of the 5954 DMRs in phase 1, and 68% of the 6389 DMRs in phase 3 (Fig. 3). DMRs were mapped to genes and gene ontology (GO) terms were slimmed to identify the least dispensable significant (*p* < 0.05) terms. Across all phases, when examining the effect of *DNMT3L* overexpression, these slimmed GO terms represented different neurodevelopmental processes, including synapse, signaling, and biological adhesion. However, there were also phase-specific GO terms, including the glucuronidation hierarchy of GO terms in the phase 1 comparison. While represented by xenobiotic glucuronidation, the glucuronidation hierarchy appears to represent retinoic acid, which is added to the media in phase 1, since the glucuronidation of retinoic acid is involved in neurodevelopment [25].

**Fig. 4:**
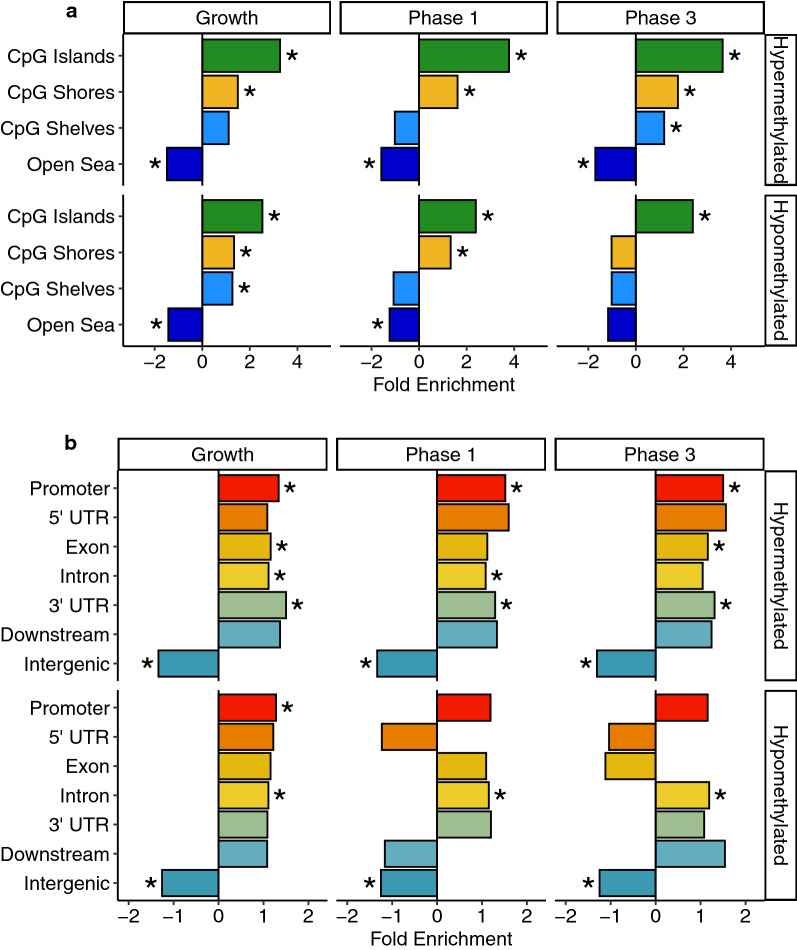
Annotation enrichments for DMRs from the growth, phase 1, and phase 3 comparisons. **a** CpG and **b** genic annotation enrichments for hypermethylated and hypomethylated DMRs. * = *q* < 0.05

To further test the functional relevance of DNA methylation changes resulting from *DNMT3L* overexpression, we tested the DMRs for enrichments within genomic regions annotated as CpG islands, CpG shores, CpG shelves, or open sea, as well as for enrichments within promoter and gene body annotations. Both the hypermethylated and hypomethylated DMRs from all pairwise phase comparisons were significantly (*q* < 0.05) enriched within CpG islands (Fig. 4a) and significantly de-enriched within intergenic regions (Fig. 4b). Furthermore, the hypermethylated DMRs from all pairwise phase comparisons were significantly (*q* < 0.05) enriched within promoters and regions of the gene body; while, the hypomethylated DMRs from all pairwise phase comparisons were significantly (*q* < 0.05) enriched within introns.

**Fig. 5:**
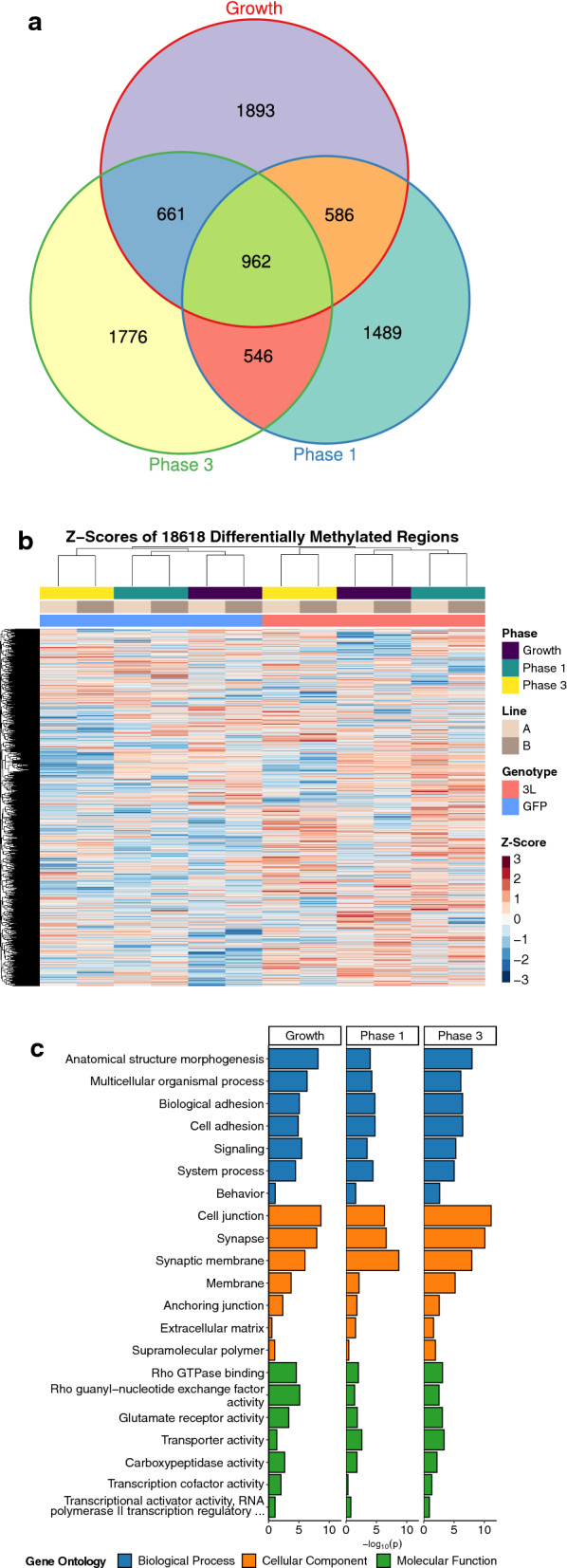
Consensus DMR profiles for *DNMT3L* overexpression across all phases of neural differentiation. **a** Euler diagram of gene symbol overlaps for the genotype comparison at each phase of neural differentiation. **b** Heatmap of hierarchal clustering of Z-scores for the consensus DMRs that are derived from merging the DMRs from each phase comparison by sequence overlap. **c** Bar plot of comparison specific p-values from the meta-analysis of the least dispensable significant slimmed GO enrichments (*p*_meta_ < 0.05)

To understand the similarities and differences between the impact of *DNMT3L* overexpression on each developmental phase, DMRs from all pairwise comparisons were compared for overlap by both genomic coordinate and gene association. Overlaps of the DMRs by gene symbol included 962 in common to all three phases (Fig. 5a), even though only 12 overlapped by genomic coordinate in all three phases. The DMRs from all phase comparisons were then merged by sequence overlap to produce a consensus DMR profile, and hierarchal clustering analysis revealed that the cells clustered primarily by genotype and then developmental phase, and not by cell line of origin (Fig. 5b). The consensus DMR profile highlighted not only the overall skew towards DMR hypermethylation, but also clusters of DMRs that were specific to differentiation phase. A meta p-value of analysis of the GO terms from all pairwise phase comparisons revealed that the least dispensable significant (*p* < 0.05) terms were largely similar to the individual comparisons and primarily represented neurodevelopment (Fig. 5c).

### The hypermethylated DMRs are enriched for bivalent chromatin and DS cross-tissue regions

**Fig. 6:**
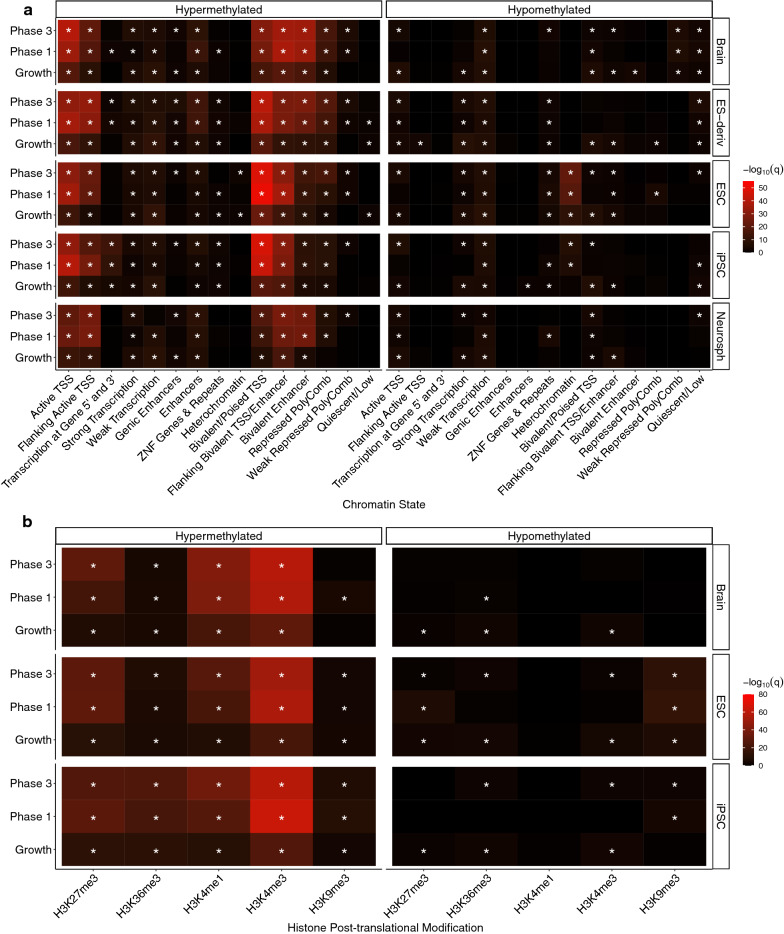
Reference epigenome enrichment analyses for the hypermethylated and hypomethylated DMRs from the genotype comparisons at each phase of neural differentiation. **a** Summary heatmap of top *q*-values for the chromHMM core 15-state enrichment analyses for the brain, embryonic stem cell derivatives (ES-derivatives), embryonic stem cells (ESC), induced pluripotent stem cells (iPSC), and neurospheres (Neurosph) categories. **b** Summary heatmap of *q*-values for Roadmap epigenomics 127 reference epigenomes 5 core histone modification enrichment analyses for the Brain, ESC, and iPSC categories. All enrichments are relative to background regions. * = *q* < 0.05

To further investigate the functional relevance between the hypermethylated and hypomethylated *DNMT3L* DMRs, enrichment analyses within chromatin state maps from the reference epigenomes were performed. The most significant (*q* < 0.05) chromHMM chromatin state enrichments overall were for the hypermethylated DMRs in regions of bivalent chromatin (Fig. 6a) marked by H3K4me3 (Fig. 6b) in stem cells. This effect was most pronounced in the differentiated cells. In contrast, the hypomethylated DMRs were most significantly (*q* < 0.05) enriched within heterochromatic regions (Fig. 6a) marked by H3K9me3 (Fig. 6b) in ESCs.

**Fig. 7:**
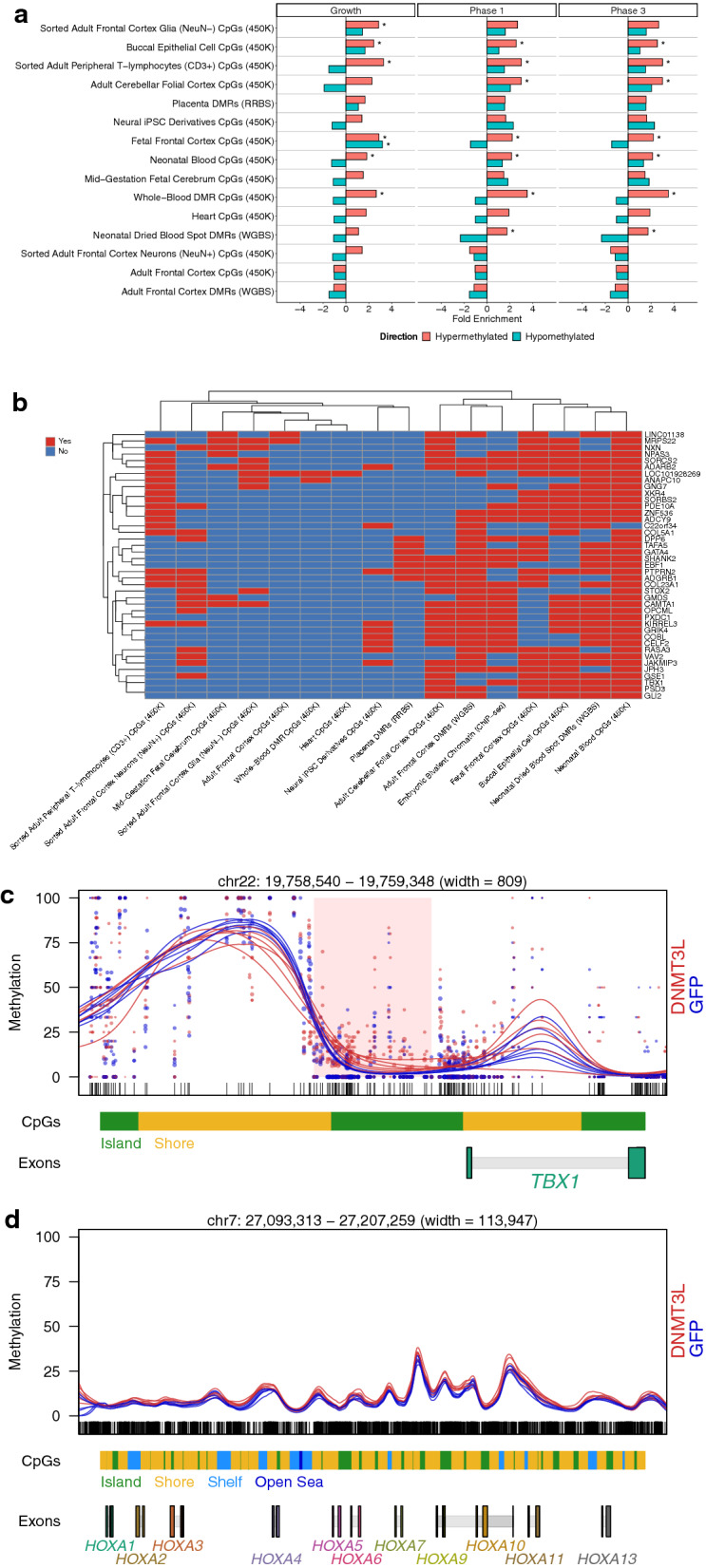
Enrichments of previously reported Down syndrome differentially methylated loci among the *DNMT3L* overexpression DMRs at each phase of neural differentiation. **a** Bar plots of enrichments of Down syndrome differentially methylated loci among the *DNMT3L* DMRs, shown for each of the available Down syndrome methylation datasets. The enrichments are relative to background regions. * = *q* < 0.05. **b** Heatmap (hierarchal clustering) of pan-tissue Down syndrome differentially methylated genes (DMRs in at least 5 datasets from previous studies), color coded for concordance or lack of concordance with the differential methylation produced by DNMT3L overexpression in SH-SY5Y neuroblastoma cells. Intergenic mappings were excluded. **c** Plot of the *DNMT3L* overexpression DMRs within the *TBX1* promoter region. Samples at each phase of neural differentiation have been color coded by genotype. The dots represent the methylation value of a single CpG site and their size represents coverage, while the lines represent an estimate of the individual methylation value for a sample. **d** Plot of hypermethylation of the *HOXA* cluster in *DNMT3L*-overexpressing cells

To test the hypothesis that the *DNMT3L* DMRs represent a facet of the differentially methylated genes observed in DS, cross-tissue and pan-tissue comparisons with differentially methylated DS sites across diverse tissues were performed [26–35]. These results revealed a significant (*q* < 0.05) enrichment of the *DNMT3L* DMRs within DS differentially methylated sites identified from multiple tissues (Fig. 7a). This enrichment was predominantly for the hypermethylated *DNMT3L* DMRs from all pairwise phase comparisons. The only significant (*q* < 0.05) enrichment for the hypomethylated *DNMT3L* DMRs was from the growth comparison and within fetal frontal cortex CpGs. To further understand the DS differentially methylated genes most impacted by DNMT3L, genes with *DNMT3L*-associated differential methylation in all three phases of neural differentiation were filtered to include those also differentially methylated in at least 5 DS methylome datasets, and also investigated for overlap with embryonic bivalent chromatin marks from a previous study [36] (Fig. 7b). In this analysis, the common pan-tissue genes mapping to the *DNMT3L*-specific DMRs were generally from perinatal or neuronal DS datasets and most also mapped to bivalent chromatin. Notably, in the *DNMT3L* overexpression cells lines, one of the pan-tissue DMR sites identified maps to the promoter of *TBX1* (Fig. 7c). *TBX1* is a transcription factor involved in embryonic development and a key gene in 22q11.2 deletion syndrome (DiGeorge syndrome). DiGeorge syndrome shares phenotypic similarities with DS, which include congenital facial and cardiac abnormalities as well as intellectual disability [37]. Additionally, *TBX1* is regulated by retinoic acid, which is a key component of the SH-SY5Y differentiation protocol utilized [23].

Also present amongst the *DNMT3L* differentially methylated genes were subsets of the 25 previously known pan-tissue and multi-tissue DS genes [2], which were all hypermethylated. This observation is in line with the previous observation that 24 out of the 25 pan-tissue and multi-tissue DS genes are hypermethylated. The growth comparison contained DMRs mapping to *ZNF837*, *RYR1*, *BCL9L*, *VPS37B*, and *RUNX1*. The phase 1 comparison contained a DMR mapping to *TEX14*. The phase 3 comparison contained DMRs mapping to *BCL9L*, *RFPL2*, *RYR1*, *ADAMTS10*, *MZF1*, and *RUNX1*.

The *DNMT3L* DMRs also mapped to two clusters of genes previously known to be altered in DS and important in neurodevelopment, specifically the protocadherin and *HOX* genes clusters [2]. The clustered protocadherin locus is located on chromosome 5 and involved in the establishment of single-cell neuronal identity [38]. Notably, the protocadherin gamma cluster contains one of the previously known 25 pan-tissue DS genes (*PCDHGA2*) [2], and differential methylation within the cluster has been observed in diverse DS tissues, which include fetal brain, adult brain, neonatal blood, T-lymphocytes, buccal epithelial cells, and placenta [29–31, 33–35]. The protocadherin gamma cluster contained two hypermethylated *DNMT3L* DMRs and one hypomethylated DMR in the phase 1 comparison. The protocadherin beta cluster contained a hypermethylated *DNMT3L* DMR in the growth comparison and a different hypermethylated DMR in the phase 3 comparison. The clustered *HOX* genes, which are involved in early developmental patterning, contained *DNMT3L* DMRs in all phase comparisons. The *HOXA* gene cluster also contains one of the previously known 25 pan-tissue genes (*HOXA4*) [2], and large-scale hypermethylation of the cluster was apparent in *DNMT3L*-overexpressing cells at all phases of differentiation (Fig. 7d). Differential methylation of the *HOX* clusters has been observed in a number of tissues, which includes adult brain, fetal brain, neonatal blood, whole-blood, T-lymphocytes, placenta, buccal epithelial cells, and neural induced pluripotent stem cell (iPSC) derivatives [26, 27, 30–35]. *DNMT3L* DMRs mapping to *HOXB7*, *HOXC4*, *HOXC5, HOXD10*, and *HOXD12* were present in the growth comparison, while those mapping to *HOXD-AS2*, *HOTAIR* (“HOX transcript antisense RNA” for the *HOXC* cluster), *HOXC5*, *HOXC6*, *HOXC12*, and *HOXC13, and HOXD12* were present in the phase 1 comparison, and those mapping to *HOTTIP* (“*HOXA* distal transcript antisense RNA”), *HOXC-AS2*, *HOXC-AS3*, *HOXC12*, and *HOTTAIR* were present in the phase 3 comparison. The presence of *DNMT3L* DMRs in the *HOX* gene clusters is also notable given that retinoic acid is known to regulate their expression and chromatin state in mESCs [39].

While the *DNMT3L* DMRs overlapped with many of the 106 imprinted genes in the human genome, these results were not significantly higher than expected in enrichment analyses. The growth DMRs mapped to 18 imprinted genes: *SNRPN, ZNF597, ZFAT, NTM, LIN28B, GRB10, MIMT1, KCNQ1, ADTRP, ANO1, OSBPL5, FAM50B, ATP10A, SNORD116, MIR296, GLIS3, PPP1R9A,* and *KCNK9.* The phase 1 DMRs mapped to 19 imprinted genes: *ATP10A, MKRN3, PHLDA2, RB1, MESTIT1, IGF2, DGCR6L, ZFAT, NTM, MIR296, MAGI2, ZIM2, SNORD116, KCNK9, ZDBF2, NAA60, KCNQ1, GNAS,* and *KCNQ1OT1*. The phase 3 DMRs mapped to 22 imprinted genes: *OSBPL5, TP73, MKRN3, WT1, KCNK9, NAA60, PPP1R9A, ATP10A, SNRPN, PLAGL1, GRB10, SNORD116, SLC22A3, MEG8, MEG3, ZIM2, MIMT1, GNAS, UBE3A, NDN, IGF2,* and *ADTRP*. This lack of enrichment is consistent with the literature, as although DNMT3L is required for the establishment of imprinting, it is not required for its maintenance [14].

Finally, we examined the effect of lot-to-lot variation on *DNMT3L* overexpression in cell culture. This involved generating an additional set of replicate SH-SY5Y cell lines from a more recent lot of cells than the one used for the first two sets of transfection experiments. While this additional set of cell lines clustered differently from the other two and showed a stronger hypermethylation profile in *DNMT3L*-overexpressing cells compared to control cells, a replication analysis that included all three transfection experiments still reproduced the main the findings (Additional File 1: Supplementary Table 2 and Additional File 2: Supplementary Note and Supplementary Figures S1–S7). Together, these results demonstrate the reproducibility of the main findings of this study, which is that *DNMT3L* overexpression results in hypermethylation of genes differentially methylated in DS and regions of bivalent chromatin.

## Discussion

The results of this study confirmed previous observations and provided novel findings that are relevant to understanding the role of *DNMT3L* in establishing DNA methylation profiles during development and in DS. *DNMT3L* overexpression in undifferentiated, differentiating, and differentiated SH-SY5Y neurons resulted in a global increase in CpG and CpG island methylation. The DMRs mapped to genes involved in neurodevelopment, cellular signaling and gene regulation. Both *DNMT3L* genotype and cell differentiation phase were inter-related in the consensus DMR clustering, demonstrating that different regions of the same genes are affected during development. Furthermore, these results demonstrate that *DNMT3L* overexpression results in differential methylation of cross-tissue and pan-tissue sites within genes identified in 15 previous DS datasets from diverse tissues, with a majority of the pan-tissue genes mapping to bivalent chromatin. However, our results also highlight a directional dichotomy in the DNA methylation changes associated with *DNMT3L* overexpression. The hypermethylated *DNMT3L* DMRs showed a cross-tissue DS profile, in contrast to the hypomethylated DMRs that showed a distinct profile related to early neurodevelopment. There were also distinct differences in the chromatin state enrichments for the hypermethylated and hypomethylated DMRs.

The hypermethylated DMRs were enriched within regions of bivalent chromatin marked by H3K4me3. Bivalent chromatin consists of regions marked with both activating H3K4me3 and repressive H3K27me3 and serves to regulate key developmental genes in ESCs by repressing them during pluripotency and poising them for rapid activation upon removal of H3K27me3 during differentiation [40, 41]. Notably, while bivalent promoters in ESCs are unmethylated or hypomethylated, upon differentiation some bivalent promoters lose H3K4me3 and gain DNA methylation [14, 42, 43]. Disruption of bivalent chromatin has been previously implicated in DS [29, 35, 44]. Since DNMT3L recognizes unmethylated H3K4 [10], a possible mechanism is that regions of bivalent chromatin lose H3K4me3 as they differentiate and become hypermethylated by the excess DNMT3L that recognizes the H3K4me0. This hypothesis is consistent with the observation that phases 1 and 3 *DNMT3L* DMRs show much stronger enrichments within regions marked by H3K4me3 when compared to the growth phase. This proposed mechanism is also consistent with the growth phase still showing enrichment within regions marked by H3K4me3, as the cells are already committed to the neural crest lineage. Additionally, while bivalent chromatin is generally hypomethylated in normal cells, in cancer cells, bivalent chromatin becomes hypermethylated and is associated with increased developmental gene expression (45, 46). In neural progenitors, many promoters with the H3K27me3 modification gain DNA methylation during differentiation [47].

In comparison to the hypermethylated DMRs, the hypomethylated *DNMT3L* DMRs in our study were enriched within regions of heterochromatin marked by H3K9me3. Notably, the observation of DNMT3L causing hypomethylation has also been previously established. Knockdown of *DNMT3L* in mESCs resulted in 14,107 regions showing a decrease in methylation and 5,724 showing a gain in methylation [13]. This ratio is comparable to our observations and the regions identified significantly (*p* = 0.0002) overlap with the genomic coordinates for the consensus *DNMT3L* DMRs. Mechanistically, DNMT3L is known to either release or sequester DNMT3A from heterochromatin, which has been hypothesized to enable preferential targeting of euchromatin [48]. Taken together, we hypothesize that in our study, the release or sequestering of DNMT3A from heterochromatin by excess DNMT3L resulted in the preferential targeting of bivalent chromatin within regions that recently lost H3K4me3 as the cells differentiated.

## Conclusions

While *DNMT3L* overexpression during neuronal differentiation recapitulates an important facet of the epigenetic signature of DS, it is not the only mechanism. The other methylation differences observed in DS brain are likely related to age, medication exposure, and an increase in copy number of the other genes encoding proteins related to DNA methylation and one-carbon metabolism that are also located on chromosome 21 [2]. For example, we observed hypermethylation of the chromosome 21 *RUNX1* locus in both the growth phase and phase 3 cells. Previously, we observed hypermethylation of *RUNX1* as well as genome-wide hypomethylation of *RUNX1*-binding sites in neonatal DS when compared to non-DS control blood [35]. However, we did not observe hypomethylation of *RUNX1*-binding sites in *DNMT3L*-overexpressing cells, likely because *RUNX1* was diploid in SH-SY5Y cells. The results of our study also demonstrate reproducibility in gene ontologies and chromatin states impacted by *DNMT3L* overexpression, despite variation inherent to individual cell lines. Overall, our findings suggest that *DNMT3L* overexpression during neuronal differentiation recreates a facet of the aberrant Down syndrome DNA methylation signature by targeting specific chromatin states that regulate genes important for neurodevelopment.

## Methods

### Plasmids and cloning

*DNMT3L* was cloned into an expression plasmid by Gibson assembly. First, human wild-type *DNMT3L* cDNA (IMAGE clone 1541874) was PCR amplified from pD3LMyc [4]. PCR amplification primers were design with the complementary overhangs necessary for Gibson assembly using NEBuilder (New England Biolabs). The sequence for the forward primer was: TGTCTCATCATTTTGGCAAAATGGAGCAGAAGCTGATCTCAGAGGAGGAC. The sequence of the reverse primer was: TCACCGCATGTTAGCAGACTTCCTCTGCCCTCGCCACCTCCGCTGCCGCCTAAAGAGGAAGTGAGTTCTGTTGAAAAATACTTG. The reverse primer was designed to include a sequence coding for a self-cleaving P2A peptide inframe of DNMT3L. A PiggyBac-compatible expression plasmid, PbCAG-eGFP, was a gift from Joseph Loturco (Addgene plasmid # 40973) [24]. PbCAG-eGFP was linearized with EcoRI. Both the linearized plasmid and the DNMT3L PCR amplicon were run on an agarose gel and purified in a silica column (Qiagen). Purified fragments were mixed at 1:3 molar ratio and assembled with NEBuilder HiFi DNA Assembly Mater Mix (New England Biolabs) according to the manufacturer’s instructions. Assembled products were then introduced into NEB Turbo E. coli competent bacteria by chemical transformation, plated on agar plates supplemented with ampicillin, and grown at 37 °C according to the manufacturer’s instructions. Colonies were then grown in LB and the plasmid was purified in spin silica columns according to manufacturer’s instructions. Resulting pb-DNMT3L-P2A-eGFP plasmids were confirmed by Sanger sequencing before proceeding to cell culture transfection experiments.

### Cell culture and transfection

Low passage SH-SY5Y cells in the undifferentiated growth phase were transfected with the plasmids via Lipofectamine 3000 (Invitrogen) according to the manufacturer’s instructions. After transfection, the cells were grown to a previously established timepoint when transient expression was shown to dissipate, so that only cells with stable integration could be sorted for GFP via flow cytometry. After flow cytometry, the population of > 95% GFP-positive cells were re-cultured, expanded for ~ 5 passages, and then differentiated according to Shipley et al*.* [23] except that the newer B-27 plus neuronal cell culture system (Gibco) was used and the retinoic acid was dissolved in DMSO. DNA was isolated from flash frozen cell pellets using the QIAamp DNA Micro kit (Qiagen) according to the manufacturer’s instructions.

## Western Blot

Protein was prepared by homogenizing cells in RIPA buffer (Alfa Aesar) and quantitated by Bicinchoninic Acid (Thermo Fisher) following the manufacturers' instructions. Ten micrograms of protein per sample were loaded on Bis–Tris gel (Thermo Fisher Scientific) and transferred to a PVDF membrane (Bio-Rad) according to the manufacturers' instructions. Immunoblotting was visualized on a LiCor Odyssey instrument. Antibodies for immunoblotting were: DNMT3L (Santa Cruz Biotechnology, Catalog # sc-393603) and GAPDH (Advanced ImmunoChemicals Inc., Catalog # 2-RGM2). Molecular weights were determined using known weight markers and reported weights.

## WGBS

WGBS library preparation was performed using the post-bisulfite adaptor tagging (PBAT) method with the terminal deoxyribonucleotidyl transferase-assisted adenylate connector-mediated single-stranded-DNA ligation technique [49, 50] via the Accel-NGS Methyl-Seq DNA Library Kit (Swift Biosciences) with the Methyl-Seq Combinatorial Dual Indexing Kit (Swift Biosciences) according to the manufacturer’s instructions. The library pool was sequenced across 2 lanes on an Illumina NovaSeq 6000 S4 flow cell for 150 bp paired end reads to generate ~ 150 million unique read-pairs (~ 10X coverage) of the genome per sample.

### Bioinformatic analyses

Trimming of adapters and methylation bias, screening for contaminating genomes, alignments to hg38, deduplication, calculation of coverage and insert size metrics, extraction of CpG methylation values, generation of genome-wide cytosine reports (CpG count matrix), and examination of quality control metrics were performed using CpG_Me (https://github.com/ben-laufer/CpG_Me) [35, 51–53]. DMR calling as well as most downstream analyses were performed using DMRichR, which utilizes the dmrseq and bsseq algorithms (https://github.com/ben-laufer/DMRichR) [35, 54, 55]. Linear mixed-effects models of the average methylation level were utilized for global and CpG island methylation level analyses, where genotype (*DNMT3L* or *eGFP*) and cellular differentiation phase were direct effects, while cell line of origin was a random effect. Transcription factor motif enrichment testing using HOMER [56], DS cross-tissue enrichment testing via GAT [57], as well as enrichment testing for histone post-translational modifications (5 marks, 127 epigenomes) and chromatin states (15-state chromHMM model) [58, 59] through LOLA [60], were all performed as previously described [35].

## Supplementary Information

**Additional file 1**: **Table 1**. Testable background regions and significant DMRs for DNMT3L overexpression in A) growth phase, B) phase 1, and C) phase 3. **Table 2**. Testable background regions and significant DMRs for DNMT3L overexpression in all 3 cell line replicates for A) growth phase, B) phase 1, and C) phase 3 (XLSX 78248 KB)

**Additional file 2**: Supplementary note. **Figures S1–S7**. Additional replicate cell line set analysis (PDF 10503 KB)

## Data Availability

The datasets generated and/or analyzed during the current study are available in the NCBI Gene Expression Omnibus (GEO) repository, through accession number: GSE168276.

## Abbreviations

DS: Down syndrome
DNMT3L: DNA methyltransferase 3L
DMRs: Differentially methylated regions
WGBS: Whole genome bisulfite sequencing
ADD: ATRX–DNMT3–DNMT3L
mESCs: Mouse embryonic stem cells
eGFP: Enhanced green fluorescent protein
PCA: Principal component analysis
iPSC: Induced pluripotent stem cell
