# Supplementary material for: Stable DNMT3L overexpression in SH-SY5Y neurons recreates a facet of the genome-wide Down syndrome DNA methylation signature
Source: Epigenetics Chromatin. 2021 Mar 9;14:13. doi: 10.1186/s13072-021-00387-7 (PMC7942011; doi:10.1186/s13072-021-00387-7)
Supplement: Supplementary file 2 — Additional file 2: Supplementary note. Figures S1–S7. Additional replicate cell line set analysis (PDF 10503 KB) [file 13072_2021_387_MOESM2_ESM.pdf]

Supplementary Note

To test the reproducibility of the observed effects on DNMT3L expression on genome-wide DNA methylation, we carried out a replication analysis using a separate lot of SH-5YSY cells. Like the two primary replicate sets in our study, the third replicate set was also derived from low passage cells from ATCC. DNMT3L overexpression was confirmed in the third replicate (**Supplementary Figure 1**). The main analyses were then repeated to include the third replicate set. Similar to our original observation, there was still a significant (p = 0.002) increase in global CpG methylation (**Supplementary Figure 2A**) and a significant (p = 0.0008) increase in CpG island methylation from 3L overexpression. Phase had a significant (p = 0.01) effect on global CpG methylation but, in contrast to the previous analyses, there was no significant (p = 0.25) effect of phase on global CpG island methylation. Similar to the other analyses, PCA revealed that the cells clustered together by phase for both the 20 Kb window and single CpG approaches (**Supplementary Figure 2B**), but clustered by genotype for the CpG island approach (**Supplementary Figure 2C**). When the third replicate set was included in the DMR calling (**Additional File 1: Supplementary Table 2**), there was a stronger hypermethylation skew: 75% of the 3359 growth DMRs, 86% of the 4885 phase 1 DMRs, and 91% of the 7279 phase 3 DMRs (**Supplementary Figure 3**). The GO terms were also similar and the DMRs mapped to genes involved neurodevelopment, cellular signaling, and gene regulation (**Supplementary Figure 3**). The significant (q < 0.05) enrichment of the hypermethylated DMRs within CpG islands was also replicated; however, the hypomethylated DMRs were not significantly enriched for within CpG islands (**Supplementary Figure 4A**). Similar to the previous comparisons of just the two replicates, the hypermethylated DMRs from all pairwise comparisons were significantly (q < 0.05) enriched within promoters and regions of the gene body, while the hypomethylated DMRs from all pairwise comparisons were significantly (q < 0.05) enriched within introns (**Supplementary Figure 4B**).

From all of the pairwise phase comparisons, 711 of the DMRs overlapped by gene symbol (**Supplementary Figure 5A**) and 50 overlapped by sequence. In the consensus DMR heatmap, the 3rd replicate clustered closer with itself than by genotype, suggesting that the primary differences are due to the characteristics of the cell line before transfection (**Supplementary Figure 5B**). However, a heatmap of the consensus DMRs with just the 3rd replicate set showed the expected clustering by differentiation timepoint (**Supplementary Figure 5B**). The meta p-value GO analysis also showed similar terms related to neurodevelopment, cellular signaling, and gene regulation (**Supplementary Figure 5C**).

The chromHMM chromatin state and histone modification enrichments also replicated, although with a larger effect size. The hypermethylated DMRs were significantly (q < 0.05) enriched in regions of bivalent chromatin (**Supplementary Figure 6A**) marked by H3K4me3 (**Supplementary Figure 6B**) in stem cells, an effect most pronounced in the differentiated cells. The hypomethylated DMRs were most significantly (q < 0.05) enriched for within regions heterochromatin (**Supplementary Figure 6A**) marked by H3K9me3 (**Supplementary Figure 6B**) in ESCs. While the DS cross-tissue analysis also showed more significant (q < 0.05) enrichments for the hypermethylated DMRs in the replication analysis, the hypomethylated fetal cerebral cortex enrichment was no longer present in the growth comparison (**Supplementary Figure 7A**). The analysis of common pan-tissue genes mapping to the DNMT3L-specific DMRs showed a similar

general trend for the perinatal or neuronal datasets (**Supplementary Figure 7B**) and included a DMR in the *TBX1* promoter (**Supplementary Figure 7C**). DMRs mapping to the protocadherin and *HOX* gene clusters were also present (**Supplementary Figure 7D**). Overall, the 3rd replicate set clustered differently from the other two sets and had a more pronounced hypermethylation profile; however, it still replicated the main conclusions of the primary analyses.

Supplementary Figures 1-7 and Supplementary Table 2 Legend

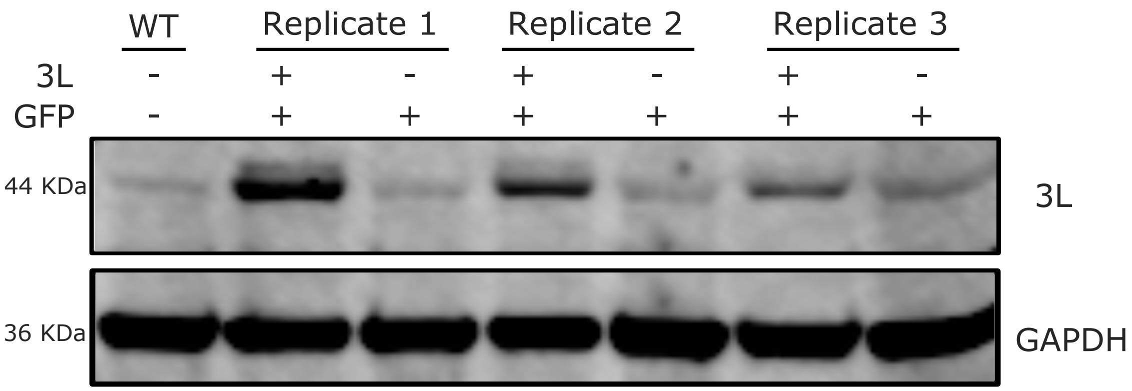

Supplementary Figure 1: Western blots of DNMT3L and GAPDH in wild-type SH-SY5Y cells (WT) and all 3 cell line replicate sets.

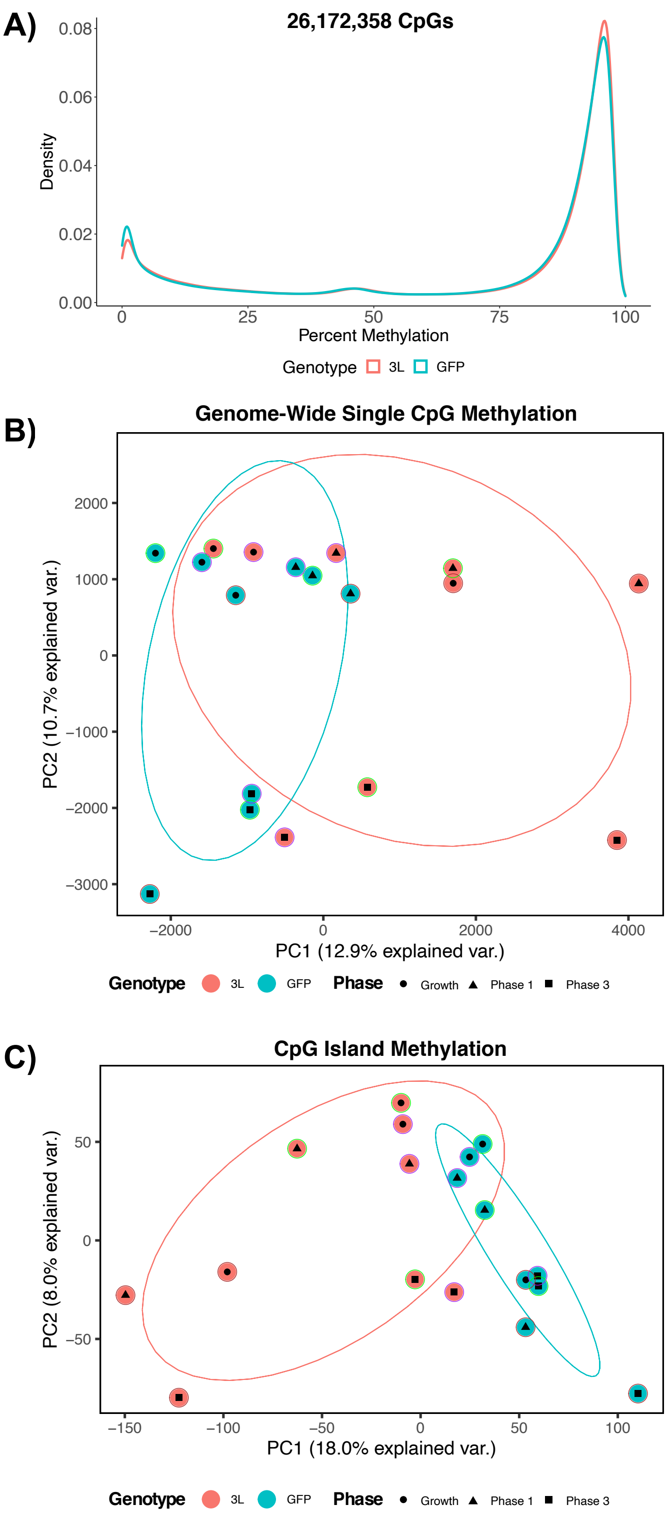

Supplementary Figure 2: Global methylation profiles of *DNMT3L* overexpression for all 3 cell line replicate sets. **A)** Density plots of the mean of smoothed individual CpG methylation values for each cell line at the different phases. **B)** Principal component analysis (PCA) of smoothed individual global CpG methylation values. **C)** PCA of smoothed individual CpG island methylation values. For each PCA, the color of the outermost shape represents the cell line, where green represents the first batch of cell lines and purple represents the second. The ellipses represent the 68% confidence interval, which represents 1 standard deviation from the mean for data with a normal distribution.

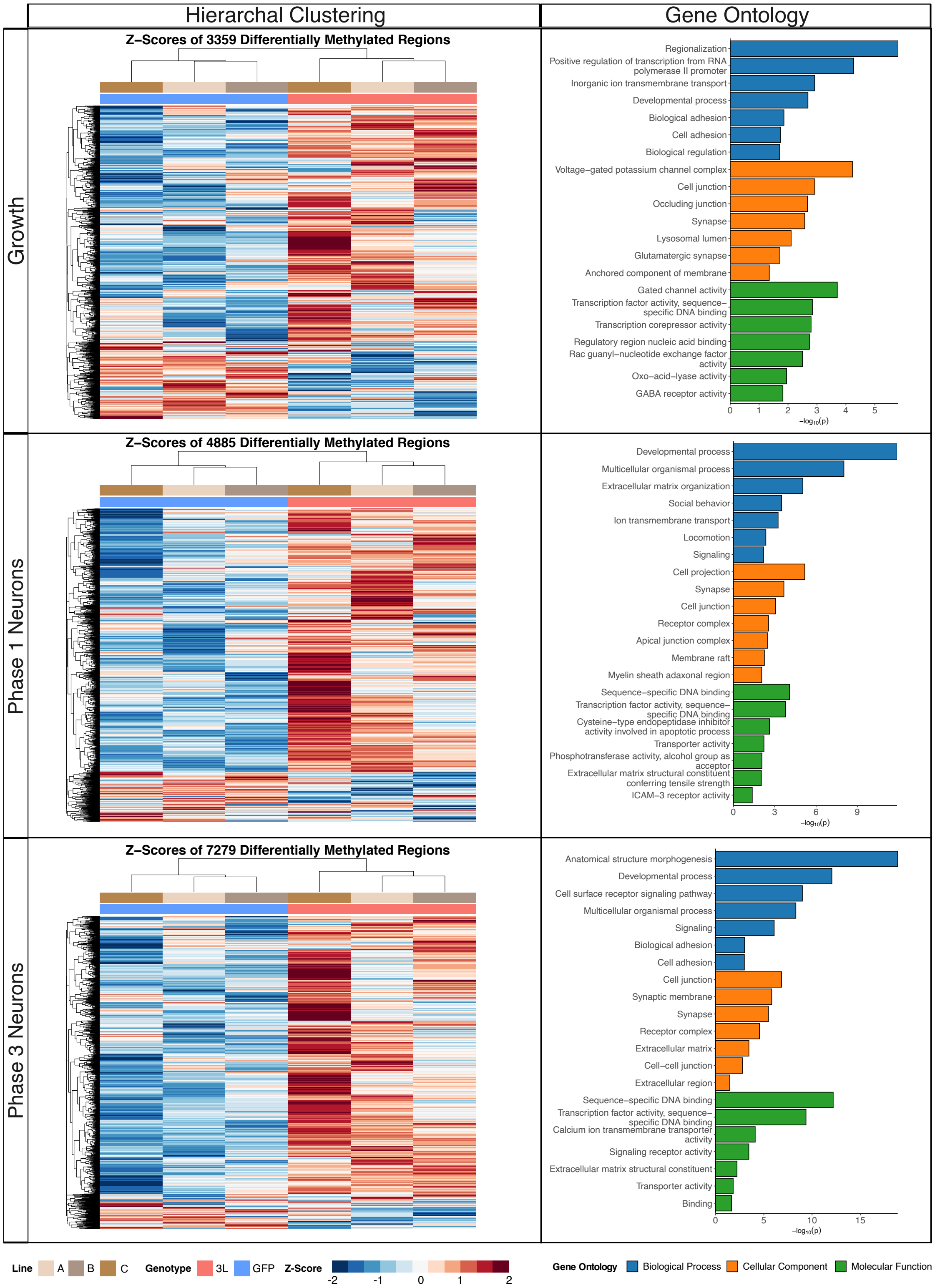

Supplementary Figure 3: Differentially methylated region (DMR) heatmaps and slimmed gene ontology (GO) enrichments for *DNMT3L* overexpression at the different phases of neural differentiation for all 3 cell line replicate sets.

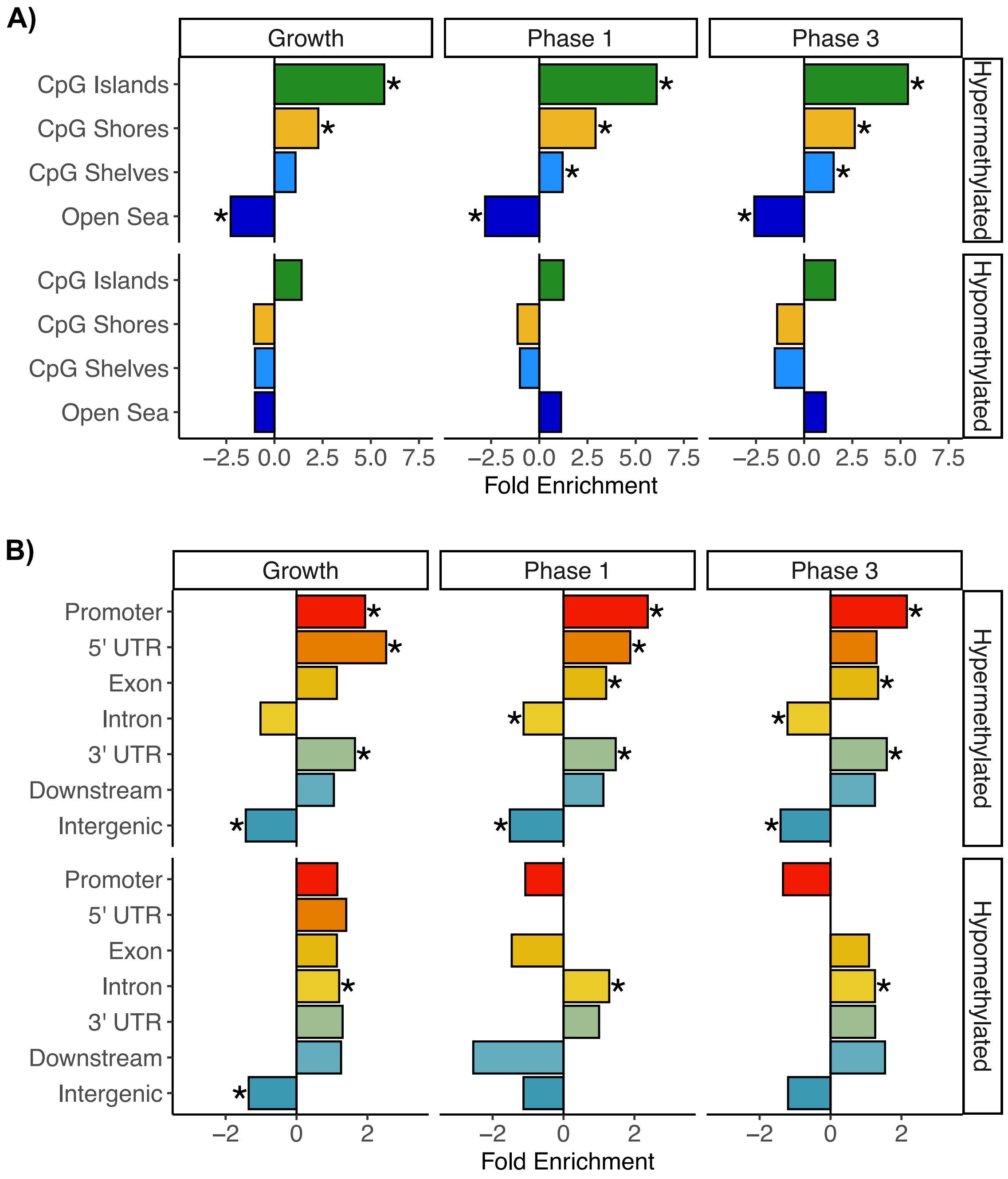

Supplementary Figure 4: Annotation enrichments for DMRs from the growth, phase 1, and phase 3 comparisons for all 3 cell line replicate sets. **A)** CpG and **B)** genic annotation enrichments for hypermethylated and hypomethylated DMRs. * = $q < 0.05$.

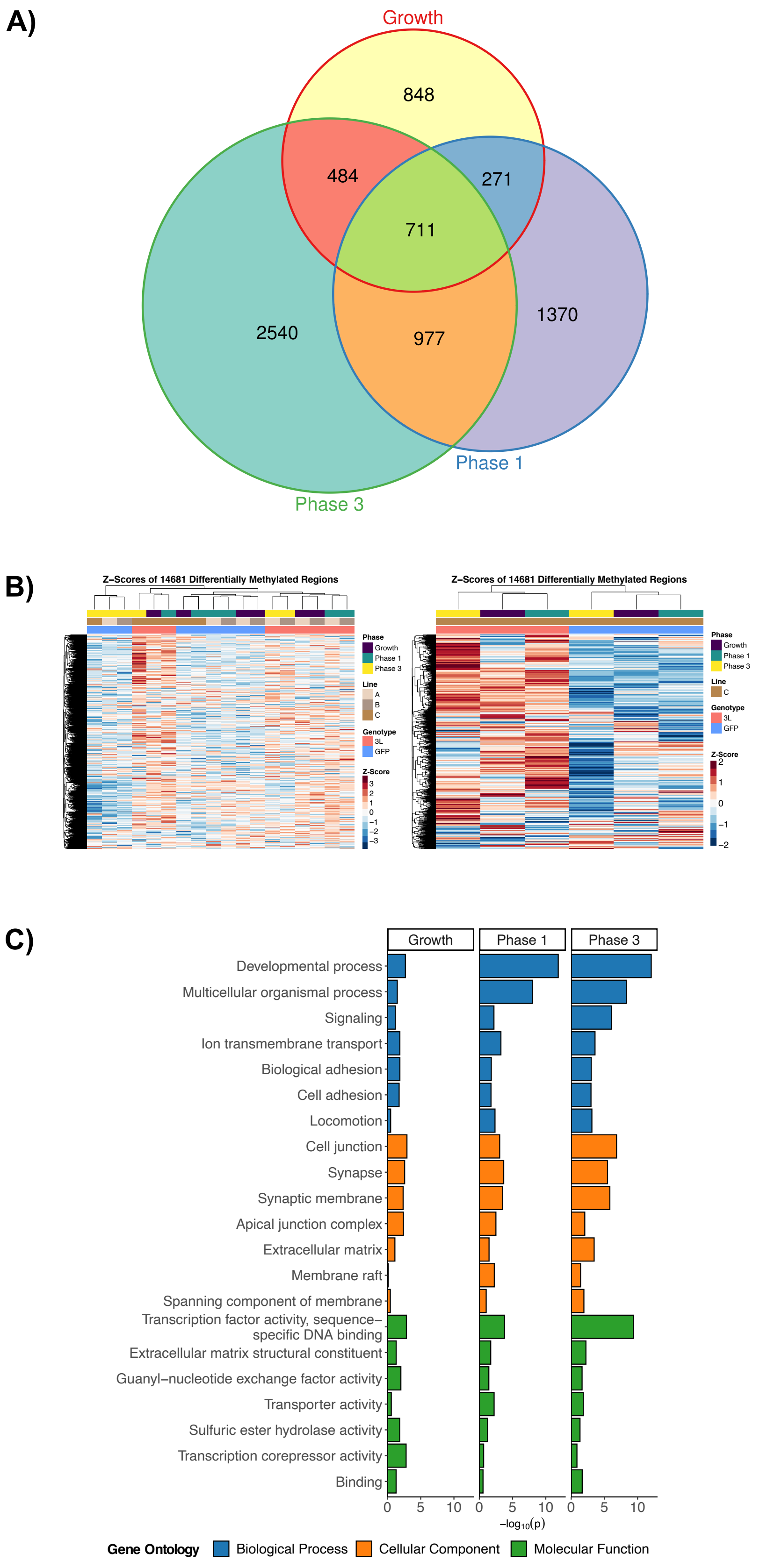

Supplementary Figure 5: Consensus DMR profiles for *DNMT3L* overexpression across all phases of neural differentiation for all 3 cell line replicate sets. **A)** Euler diagram of gene symbol overlaps for the genotype comparison at each phase of neural differentiation. **B)** Heatmaps of hierarchal clustering of Z-scores for the consensus DMRs that are derived from merging the DMRs from each phase comparison by sequence overlap. **C)** Bar plot of comparison specific p-values from the meta-analysis of the least dispensable significant ($p < 0.05$) slimmed GO enrichments.

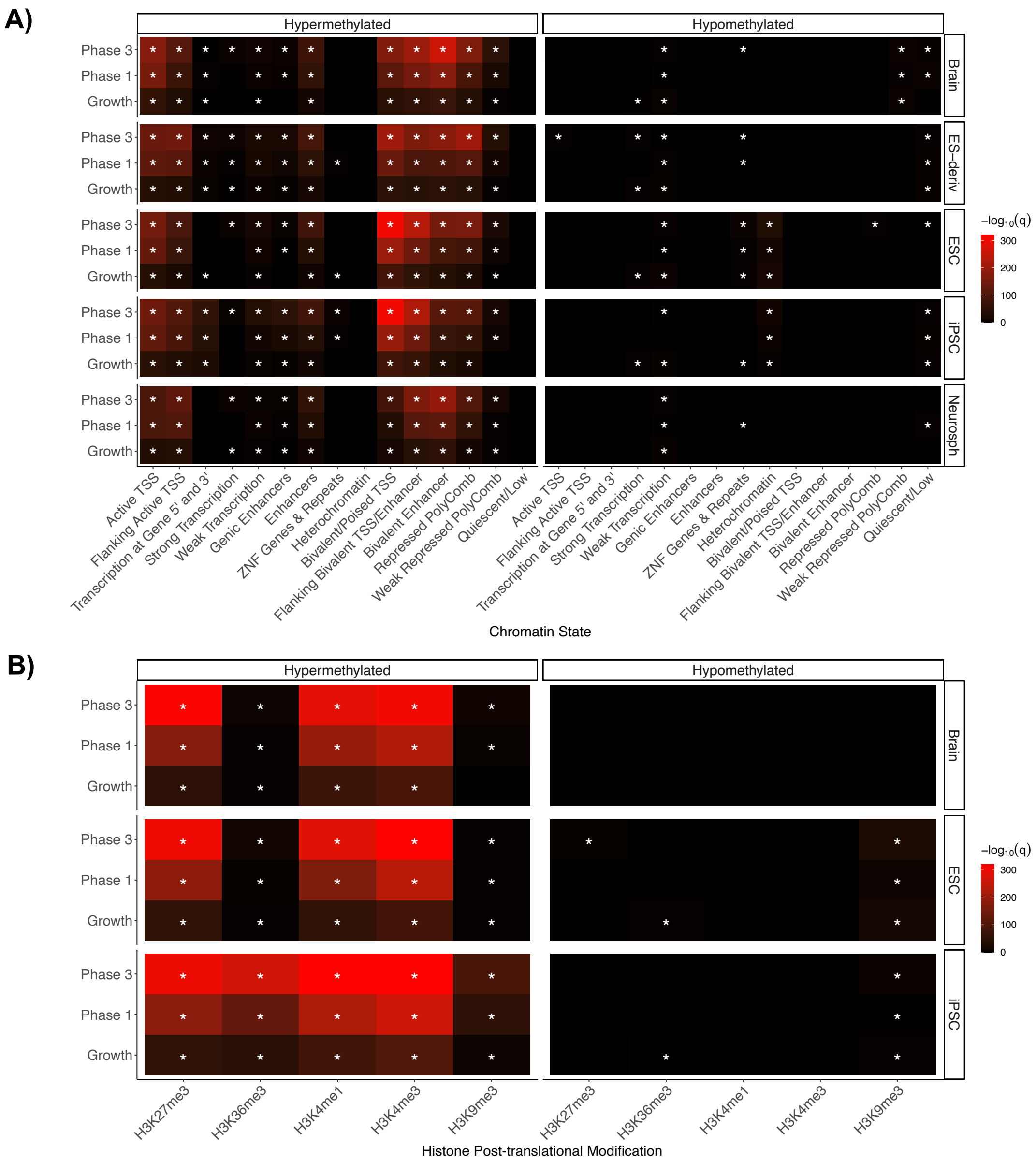

Supplementary Figure 6: Reference epigenome enrichment analyses for the hypermethylated and hypomethylated DMRs from the genotype comparisons at each phase of neural differentiation for all 3 cell line replicate sets. **A)** Summary heatmap of top *q*-values for the chromHMM core 15-state enrichment analyses for the brain, embryonic stem cell derivatives (ES-derivatives), embryonic stem cells (ESC), induced pluripotent stem cells (iPSC), and neurospheres (Neurosph) categories. Values of infinity have been replaced with the top value. **B)** Summary heatmap of *q*-values for Roadmap epigenomics 127 reference epigenomes 5 core histone modification enrichment analyses for the Brain, ESC, and iPSC categories. Values of infinity have been replaced with the top value. All enrichments are relative to background regions. * = $q < 0.05$.

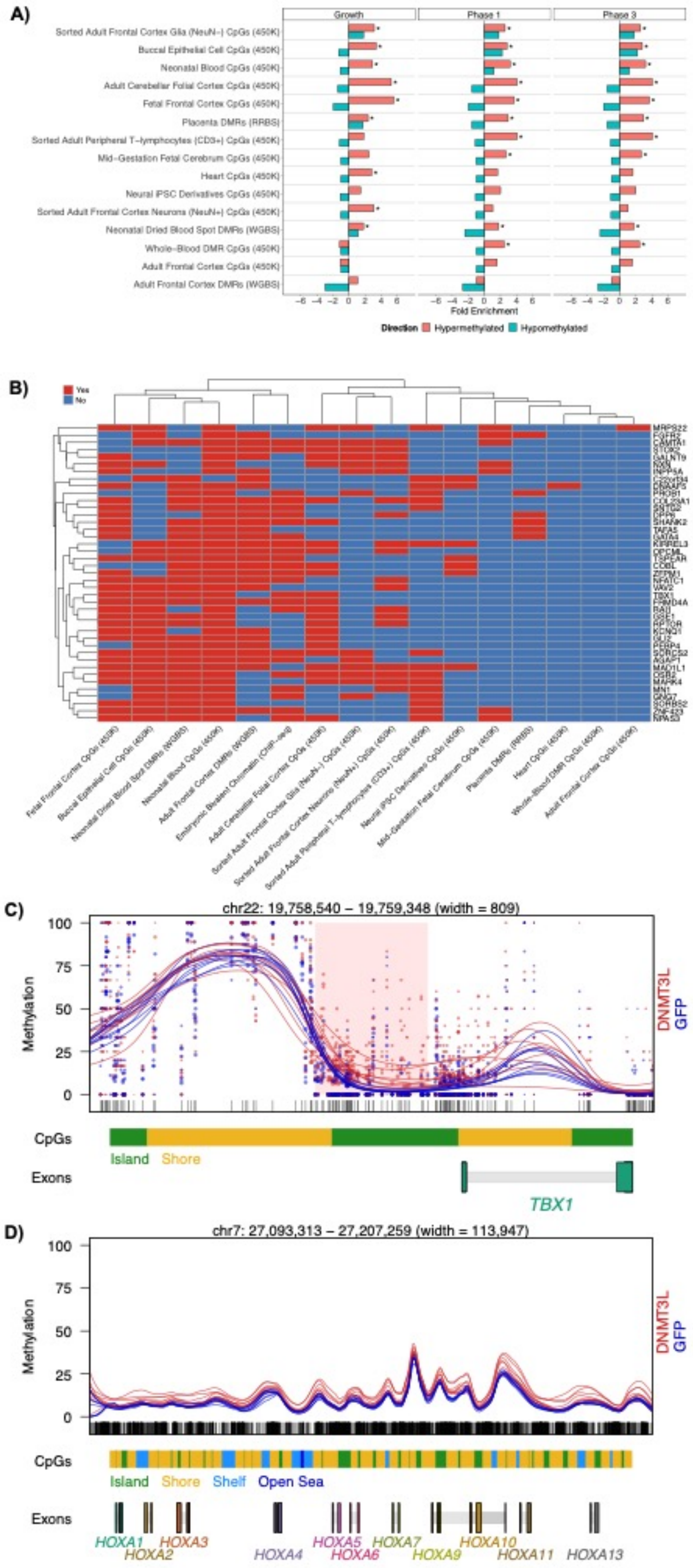

Supplementary Figure 7: Enrichments of previously reported Down syndrome differentially methylated loci among the DNMT3L over-expression DMRs at each phase of neural differentiation for all 3 cell line replicate sets. **A)** Bar plots of enrichments of Down syndrome differentially methylated loci among the DNMT3L DMRs, shown for each of the available Down syndrome methylation datasets. The enrichments are relative to background regions. * = $q < 0.05$. **B)** Heatmap (hierarchal clustering) of pan-tissue Down syndrome differentially methylated genes (DMRs in at least 5 datasets from previous studies), color-coded for concordance or lack of concordance with the differential methylation produced by DNMT3L over-expression in SH-SY5Y neuroblastoma cells. Intergenic mappings were excluded. **C)** Plot of the DNMT3L overexpression DMRs within the *TBX1* promoter region. Samples at each phase of neural differentiation have been color coded by genotype. The dots represent the methylation value of a single CpG site and their size represents coverage, while the lines represent an estimate of the individual methylation value for a sample. **D)** Plot of hypermethylation of the *HOXA* cluster in DNMT3L overexpressing cells.

Related to the Supplemental Note but located within Additional file 1

Supplementary Table 2. Testable background regions and significant DMRs for *DNMT3L* overexpression in all 3 cell line replicate sets for **A)** growth phase, **B)** phase 1, and **C)** phase 3.
